# Natrum Muriaticum

**Published:** 1890-06

**Authors:** A. McNeil

**Affiliations:** San Francisco, Cal.


### NATRUM MURIATICUM.

(TABLE SALT.)

A. MCNEIL, M. D., SAN FRANCISCO, CAL.

This drug is an incontestable evidence of the efficacy of trituration and succussion in developing the medicinal virtue of a drug. But some may say that common salt, when potentized, has no curative powers. I pity the patients of that man, calling himself a homœopathic physician, who, deterred either by prejudice or ignorance, has never seen cures performed by Natrum-mur. in potencies, although the patients have taken the crude salt at every meal, and usually in very large quantities, as such persons have a great craving for it in their food. Dr. Watzke, of Vienna, undertook to prove this drug, and, in spite of his preconceived opinions, was compelled to acknowledge that not till he went up to the high potencies, could he produce symptoms in the healthy of any value.

In the mental symptoms, there is a resemblance to Ignatia, as there is much inclination to weep, but there is more excitability than with that drug, in which the disposition is to be quiet and passive. Sometimes, with this sadness and weeping, the patient is much aggravated by consoling words, so that they start the heart fluttering. With Arsen. consoling words excite cough. Sometimes the Nat.-mur. patient is hypochondriacal and tired of life, having a resemblance to Nux-v.; but a glance at the concomitants of these drugs will prevent any mistake. The patient may be in a hurried frame of mind, with anxiety and fluttering of the heart.

This is one of the important remedies in headache, particularly those attending intermittents and in uterine complaints. Its headache usually begins in the morning on waking, with Bryonia. It comes on first beginning to move, and disappears on the breaking out of sweat. The headache is as if the head would burst. In this it resembles many other drugs. It cures where there is a heaviness in the back part of the head with which the eyes draw together; with Cannabis-indica this is attended by pains which shoot up the sides of the head to the temples and vertex; with Eupatorium-perf. it comes on after lying down. It is the simillimum for a headache with a throbbing as if little hammers were striking, coming on when awaking every morning; with Psorin. this throbbing is from within outwards. Many remedies have a feeling as if the head would burst on coughing; but only Natrum-m. and Staphisagria have as if the forehead would burst on coughing.

With Natrum-mur. there is a fiery zigzag appearance around all objects, while with Sepia these fiery zigzags appear before the eyes without regard to the objects looked at. Salt also cures when black spots and streaks of light appear to him. The patient may be affected with a sudden darkness, so that everything turns black. In the weakness of vision give this remedy if there is a drawing, stiff sensation in the muscles of the eyes when moving them; objects run together on exerting the eyes; this is quite similar to Ruta, and it, no doubt, has been often given and, of course, unsuccessfully, when Natrum-mur. should have been prescribed. In ophthalmia, both catarrhal and scrofulous, when Nitrate of silver has been used, always give Natrum-mur. at first and it may prove the simillimum for the entire case.

Loss of smell and taste, especially with catarrh, indicate one of two drugs, salt or Pulsatilla, but the concomitants and modalities will decide which should be used. The secretion of clear mucus, transparent, like the white of egg, from the nose, should always call your attention to Natrum-mur.

Patients, particularly ladies, often have their lives almost made miserable by the appearance of the face which is such that it looks as if it had been greased. In this condition compare Natrum-mur., Plumbum, and Thuja carefully and you will be able to cure and bring happiness to your fair patient and coin and glory to yourself. There are but few cases in which a physician can establish a reputation more certainly than by the cure of neuralgia of the face. This prevails particularly in the so-called malarial regions, and they are often the result of chronic poisoning by Quinine. It is in just these cases that our remedy is most frequently indicated. It is necessary to carefully differentiate from Arsenic in these cases. If the tongue has a covering of clear mucous slime with little frothy bubbles at the edge, Natrum-mur. is *the* remedy.

Fever blisters or cold sores, attending colds, intermittents, etc., nearly always indicate Natrum-mur. or Rhus-tox. The modalities of the latter drug will nearly always easily decide between them, although sometimes it will prove to be a difficult task, particularly when the genus epidemicus is changing from the one remedy to the other, as I once saw happen.

Children are slow in learning to talk; with Calcarea and Silicea they are slow in learning to walk. He complains much of dryness of the tongue, which is not very dry; with Mercury he complains of dryness of the tongue, although his mouth is full of saliva. He has a sensation of a hair on his tongue. A symptom that is rare and valuable is a moss-like appearance of the tongue which this drug, Lachesis, and Thuja have; with the latter, the tongue is coated with a white skin, the edges feeling raw, it then peels off in patches and leaves dark-red, sensitive places. Natrum-mur. and Rhus both have vesicles on the tongue. This symptom and that of fever blisters and cold sores will prove of invaluable importance in many diseases, notably intermittents and other fevers.

In sore throats, where they have been burned with Nitrate of silver, this is our remedy as it is in other cases where that powerful agent has been abused, and Adelman Emmet, the greatest of gynecologists, although an allopath, says many thousands of women are suffering tortures from the abuse of lunar caustic, which was not long ago the fashionable treatment in almost all cases of real or fancied womb disease.

In almost every case that requires this remedy an unusual thirst is present. This thirst is violent and unquenchable, like that so characteristic of Arsenic, but water, when drank, does not produce the gastric disturbance and vomiting which always follows when Arsenic is the remedy. Longing for bitter things the same as with Digitalis, for beer (Nux and Sulphur), for farinaceous food, for acids (Veratrum-alb.), for salt (Calc.-carb.), for oysters and fish, and an aversion to meat (Arsenic and Plumb.), and to coffee, which is also characteristic of Nux-vom.

Although Natrum-mur. is not an especial cough remedy, yet it has a cough worthy of remembrance, that is when he coughs pains in the abdominal ring extending into the testicles, as if the spermatic cord would be torn in pieces; with Zinc. the pain in coughing is in the testicles, so that he wants to hold them up with his hands.

Natrum-mur. should be given in constipation in which, after stool, the anus feels contracted (Lachesis) or torn so that it bleeds and smarts; with Nitric acid pains come during stool, as if something were torn away lasting two or three hours.

In gonorrhœa this is the remedy when there is no pain during urination, but burning and cutting afterward; with Cannabis-sativa there is burning just after micturition, although there is also a cutting which extends from the fossa navicularis to the neck of the bladder; Cantharis has cutting before and after urination from the neck of the bladder to the fossæ. Dr. Kunkel, who is the best prescriber in Germany, has found this agent of great value in gonorrhœa when it appears at a time when it is *the* epidemic remedy for the prevailing intermittents. He has also shown that it is indicated in those cases in which any disease is ameliorated by the patient's residence on the seashore. I have confirmed this observation in my own experience. In gonorrhœa, as well as other diseases in which Nitrate of silver has been abused, give Natrum-mur.

In diseases of women Natrum-mur. does a grand work. It is indicated when every morning (Bell., Nux-vom.) there is pressing and pushing toward the genitals, must sit down to prevent prolapsus. (Bell., better when standing and sitting straight.) Also a prolapsus accompanied by aching in the back, which is ameliorated by lying down. In aversion to coitus, because of dryness of the vagina, which renders it painful, Ferrum also. Some patients, on the other hand, after an embrace, feel easy and light-hearted, and afterward ill-humored. Before her menses she is low-spirited (Lycop., Puls., Stram.); during menses the sadness continues (Am.-carb., Cimic., and Puls.). The menses are too early, too long continued, and too profuse, as with Calc.-carb., Kali-carb. Morning sickness accompanied by headache on awakening in the morning, thirst, fever blisters, etc. And also when she vomits frothy, watery mucus, and in difficult labor where it progresses slowly, the pains are feeble and she is sad and full of forebodings. The physician who gives *the* indicated remedy in his obstetrical cases will seldom or never be compelled to use instruments, and will have few or no cases of ruptured perineum or cervix in his patients.

And in infants who are slow in learning to talk on account of the muscles of the tongue and larynx not developing properly, and in those debilitated by intestinal diseases, where there is general emaciation, but most perceptible in the neck, Calcarea-phos. has the same condition of those muscles. Nor in diseases of the respiratory organs, where there is expectoration of clear, transparent mucus, and in stitching pains in the chest, should we let Bryonia and Kali-carb. cause us to forget that Nat.-mur. has those pains, but, of course, with different concomitants from those remedies.

In diseases of the heart, both functional and organic, this remedy has done good work. It is indicated in fluttering of the heart, with a weak, faint feeling, aggravated by lying down. The heart symptoms are aggravated when lying on the left side the same as with Cactus. Palpitation of the heart, when the pulsations shake the body, and ache as if a pressure rose from the abdomen and compressed the heart.

Natrum-mur. and Rhus-tox. have aching in the back, relieved by lying on something hard. The patient often lies with her arm under her back in order to obtain this pressure, with both of the remedies. The relief from motion, so characteristic of Rhus, will sufficiently differentiate between them. These back pains are nearly always indicative of uterine complaints when found in women; in men they are nearly always rheumatic and, many times, malarial in both sexes, and both remedies cure malarial complaints when indicated.

Hang-nails and cracking of the skin around the nails is characteristic of this remedy only, and will guide you to its selection many times. Sometimes there is restlessness of the limbs with this remedy, so that they must be moved constantly. This is another point in which salt resembles Rhus-tox., but with the latter remedy the restlessness arises from the aggravation of the pains and stiffness, which increase more and more until he is compelled to move. With Arsenic the restlessness arises from a mental condition; he is in such a state of anguish that he cannot keep from moving, although he is so weak that it exhausts him very much. Aconite restlessness also arises from a mental condition, something like that of Arsenic, but it is better described by the word "anxiety," but there is an entire absence of the debility of that drug. The restlessness of Arnica is because the parts on which he rests are so tender and sore that he must change so as to lie on another part of his body. These causes of moving which I have delineated will, if carefully kept in mind, enable you to differentiate between these remedies when you are at a loss to decide.

When sleeping he has very vivid dreams, that seem real when he awakes, so that he thinks that it is a reality—of thirst; of robbers. He also is a somnambulist, so that he rises and sits up in the room. Phosphorus and Silicea are indicated in this condition.

The field in which this remedy has been the most useful is in intermittent and other fevers. In these you may have difficulty in deciding between this drug and Rhus-tox. Both have much aching in the head, back, and limbs; both have fever blisters on the lips, both may have violent thirst. But with the salt, while there may be restlessness compelling him to move, there is not that stiffness and aching increasing as long as he keeps still and passing off when moving a little, as with Phos. With Natrum-mur. the chill is in the morning not later than eleven, the thirst is very great, drinking often and much, but water does not disagree, as it does in a similar thirst when Arsenic is indicated; there is much headache, which comes with the chill and continues through the hot stage till sweating begins, which relieves. The cases may be recent or may have become worse by the use or abuse of Quinine—and the use of Quinine in the crude form is always abuse—and the only excuse for giving it on the part of one who calls himself a homœopathic physician is ignorance.

In the treatment of intermittents follow the advice of Hahnemann: by watching the symptoms of *all* your cases which occur during the season in making up the totality of symptoms on which you are to prescribe. By taking this enlarged view of the disease and by the exercise of a fair amount of knowledge of materia medica you will always be able to cure intermittents. I speak from eleven years' practice in a very malarious region. When I say cure I mean the administration of the potentized drug only, for the giving of crude doses of Quinine in many cases does not cure, it only suppresses the chill and thereby injures the patient.

In wasting diseases this is one of our best remedies, he loses flesh while living well; the same condition occurs with Iodine. In infants who become emaciated, particularly about the neck. Calcarea-phos. has the same condition, and you will require the other symptoms to decide which to use.

Apis and Natrum-mur. are complementary remedies, following each other well.

